# Impact of a computer system as a triage tool in the management of pulmonary tuberculosis in a HIV reference center in Brazil

**DOI:** 10.1590/0037-8682-0451-20

**Published:** 2022-08-05

**Authors:** Mariana Pitombeira Libório, Afrânio Kritski, Isabela Neves de Almeida, Pryscila Fernandes Campino Miranda, Jacó Ricarte Lima de Mesquita, Rosa Maria Salani Mota, George Jó Bezerra Sousa, Roberto da Justa Pires, Terezinha do Menino Jesus Silva Leitão

**Affiliations:** 1 Universidade Federal do Ceará, Faculdade de Medicina, Programa de Mestrado em Saúde Pública, Fortaleza, CE, Brasil.; 2 Secretaria da Saúde do Estado do Ceará, Hospital São José de Doenças Infecciosas, Fortaleza, CE, Brasil.; 3 Universidade de Fortaleza, Faculdade de Medicina, Fortaleza, CE, Brasil.; 4 Universidade Federal do Rio de Janeiro, Programa Acadêmico de Tuberculose, Faculdade de Medicina, Rio de Janeiro, RJ, Brasil.; 5 Universidade Federal de Ouro Preto, Escola de Farmácia, Departamento de Análises Clínicas, Ouro Preto, MG, Brasil.; 6 Universidade Estadual do Ceará, Programa de Pós-Graduação em Cuidados Clínicos em Enfermagem e Saúde, Fortaleza, CE, Brasil.

**Keywords:** Tuberculosis, Screening, Clinical score, HIV

## Abstract

**Background:**

The Neural Clinical Score for tuberculosis (NCS-TB) is a computer system developed to improve the triage of presumed pulmonary TB (pPTB).

**Methods:**

A study was performed with cohorts of pPTB patients cared for at a reference hospital in Northeast Brazil.

**Results:**

The NCS-TB sensitivity was 76.5% for TB diagnosis, which shortened the time from triage to smear microscopy results (3.3 to 2.5 days; *p<*0.001) and therapy initiation (6.7 to 4.1 days; *p=*0.045).

**Conclusions:**

Although the NCS-TB was not suitable as a screening tool, it was able to optimize laboratory diagnosis and shorten the time to treatment initiation.

The World Health Organization (WHO) estimated that, globally, approximately 10 million people fall ill with tuberculosis (TB) each year^1^. 

The use of information and communication technology (ICT) tools has increased in popularity since the introduction of artificial intelligence. These algorithms are implemented in many devices that people use, generating an enormous amount of data every hour. In the public health field, generated data can be used by researchers and policymakers^2^. This data links the patient to risk groups that are characterized by the information available in a dataset using neural modeling. 

This study aimed to evaluate the performance of the Neural Clinical Score for Tuberculosis (NCS-TB) in patients with presumed pulmonary tuberculosis (pPTB) in a high human immunodeficiency virus (HIV) prevalence setting.

This was a prospective pragmatic trial that aimed to evaluate the NCS-TB in the triage, diagnosis, and treatment cascade of pPTB subjects at a state reference center for HIV care in Fortaleza City in Northeast Brazil. 

There were two cohorts of presumed PTB subjects-one cohort that was analyzed prior to the use of the NCS-TB (Phase I, P1), from March to December 2011, and another using the NCS-TB for screening of pPTB subjects (Phase II, P2), from February to December 2012.

The cohorts had distinct subjects in each phase. They were recruited from the outpatient clinic, hospital wards, and emergency units of the São José Hospital of Infectious Diseases (HSJ). Patients who met the eligibility criteria were invited to participate in the study and signed an informed consent form. 

The project was approved by the Ethics Research Committee of the HSJ (protocol number 020/2011; ethical assessment number: 0001.0.042.197-11). The procedures followed were in accordance with the ethical standards of the national committee** **on** **human experimentation and in accordance with the principles of Declaration of Helsinki, 1964, as revised in 1975, 1983, 1989, 1996, and 2000.

The study population consisted of adult subjects (≥18 years) with respiratory symptoms compatible with pPTB (cough for ≥2 weeks and/or hemoptysis and/or chest radiographic abnormalities) evaluated during the study period. The exclusion criteria were current pulmonary tuberculosis (PTB) diagnosis, current use of anti-TB treatment, and pregnancy. 

PTB cases were defined as those with positive sputum smear microscopy and/or positive culture for *Mycobacterium tuberculosis* (confirmed TB) or diagnosis based on clinical-radiological criteria following the Brazilian guidelines^3^. 

Phase I - Observation (P1): During this phase, an interview was conducted by the research team and a questionnaire was completed before the medical consultation. The questionnaire included information on sociodemographics: age, sex, race, civil status, socioeconomic level, duration of education (years, including those of the head of the family), liberty privation in the past two years, smoking habits, illicit drug use, alcohol abuse, symptoms (cough, fever, sputum, hemoptysis, weight loss, night sweats, loss of appetite, and dyspnea), and clinical background details (previous tuberculosis, hospital admission, contact with PTB patients in the past two years, and HIV serology testing). 

Smoking and illicit drug use were considered positive if the patient was currently using these substances or had used them up to one year earlier. Alcoholism was evaluated using the cut-down, annoyed, guilty, and eye-opener questionnaire^4^. 

Chest radiography and smear microscopy with cultures of two sputum samples were requested from all patients, regardless of the recruitment phase. Chest radiography results were evaluated by a radiologist, and smear microscopy results were available to attending physicians.

Phase II - Intervention (P2): In this phase (P2), the pPTB questionnaire was the same as that used in P1. The only difference was that the data were collected using the NCS-TB electronic system developed by Souza Filho *et al*.^5^. The NCS-TB uses artificial neural network models for risk group identification (low, medium, and high) and for classifying patients as having active or inactive PTB. To build this decision support system, Souza Filho *et al*.^5^ used data from a cross-sectional study previously described by de Castro *et al*.^6^. In our study, data were recorded using a netbook and 3G Internet via modem.

The NCS-TB program used the following variables: cough, hemoptysis, fever, night sweats, weight loss, chest pain, smoking, dyspnea, history of hospital admission, age, and sex^5^. The pPTB patients were classified as having a high, medium, or low probability of PTB using red, yellow, and green stickers, respectively. The subjects were then referred for medical evaluation, where the attending physician used the NCS-TB’s classification to make a clinical decision. In the laboratory, the time (in days) for the sputum collection and release of the results differed depending on the classification by the NCS-TB: red, sputum collection and results were provided on the same day; yellow, sputum collection occurred on the same day, with the results provided the following day; and green, sputum collection occurred following morning, with the results available at least a day after.

## STATISTICAL ANALYSIS

The dataset was built using Microsoft Excel and the Statistical Package for Social Sciences (SPSS Inc. Chicago, IL, USA). Fisher’s exact test was used to perform the analysis of homogeneity and to test associations in contingency tables that compared sociodemographic and clinical variables. 

The Kolmogorov-Smirnov test was used to test the normality of continuous variable distributions, and the Mann-Whitney *U* test was used to compare the P1 and P2 populations in terms of the time from triage to microscopy results and the time to therapy initiation.

The positive and negative predictive values were calculated from the following prevalence rates: 1% in the family health program, 4% in the basic health unit, 10% in the emergency care unit, and 24.8% at the hospital level. 

The results were considered significant when the *p*-value was ≤0.05. The prevalence ratio and 95% confidence intervals were calculated for the variables significantly associated with the diagnosis of PTB, which were defined above (positive sputum smear microscopy and/or positive culture for *Mycobacterium tuberculosis* and/or clinical-radiological criteria). 

Sensitivity was calculated among the TB cases using the high and moderate NCS-TB classifications. Specificity was obtained among the non-TB cases using the low probability of TB or non-TB of the NCS-TB classifications. The positive and negative predictive values were calculated using the accuracy of the NCS-TB, as folows: 1% in the family health program, 4% in the basic health unit, 10% in the emergency care unit, and >20% at the hospital level. 

**FIGURE 1:**
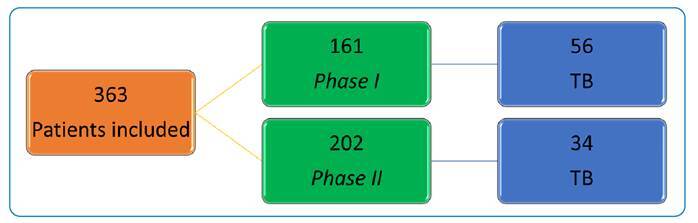
Patients with presumed tuberculosis included in Phases I and II of the study. **Legend: Phase I:** observation; **Phase II:** intervention; **TB:** tuberculosis.

The distribution of the patient samples throughout the study phases and the associated TB cases in P1 and P2 are shown in Figure 1. 

The sociodemographic characteristics of the study population according to the study phase are presented in Supplementary Material 1. The mean age of P1 and P2 was 40.3 years (SD=15.08) and 39.7 years (SD=14.7), respectively. 

Smoking, alcohol, and illicit drug abuse were variables that appeared at varying frequencies. In total, 90 patients (24.8%) were considered TB cases: 56 from baseline and 34 from implementation. All cases were tested for HIV, 18 were known to have HIV by the screening date, and 3 were diagnosed with HIV infection after inclusion in the study.

The significant variables associated with PTB cases when compared to non-PTB cases are presented in Supplementary Material 2, demonstrating the influence of social and clinical features. In comparison with the non-PTB cases, the following variables were more frequently observed among PTB cases: male sex, low education level, ≥3 weeks of productive cough, fever (≥3 weeks), night sweats, appetite loss, and absence of respiratory allergy symptoms.

The distribution of clinical variables according to the NCS-TB classification used in P2 is summarized in Table 1. For patients classified as medium or high probability, the following variables were more frequent: male sex, weight loss of 10%, fever (up to 3 weeks), blood in sputum, night sweats, and appetite loss. A few variables without information in this table were excluded from the analysis because it was assumed that their exclusion would not change the results significantly.

**TABLE 1:** Distribution of epidemiological and clinical variables according to the Neural TB Clinical Score - Phase II.

Neural TB Score Classification							
	Non-TB/Low Probability		Medium/High Probability		Total		p*
	n	%	n	%	n	%	
**Sex**							
Female	52	58.4	29	25.7	81	40.1	<0.001
Male	37	41.6	84	74.3	121	59.9	
**Race (skin color)**							
White/yellow	18	20.2	21	18.6	39	19.3	0.853
Brown/black	71	79.8	92	81.4	163	80.7	
**Marital status**							
Married/stable union	35	39.3	49	43.4	84	41.6	0.639
Divorced/separated/widower	15	16.9	14	12.4	29	14.4	
Single	39	43.8	50	44.2	89	44.1	
**Years of schooling**							
Illiterate/<4 years	19	21.3	23	20.4	42	20.8	0.213
4 to <9 years	26	29.2	43	38.1	69	34.2	
9 years	15	16.9	24	21.2	39	19.3	
≥12 years	29	32.6	23	20.4	52	25.7	
**Socioeconomic level (minimum wage)**							
≥6	10	11.2	6	5.3	16	7.9	0.239
4-5	39	43.8	47	41.6	86	42.6	
≤3	40	44.9	60	53.1	100	49.5	
**Weight loss**							
No	49	55.7	97	85.8	146	72.6	<0.001
Yes	39	44.3	16	14.2	55	27.4	
% Weight loss							
≤10%	71	80.7	55	48.7	126	62.7	<0.001
>10%	17	19.3	58	51.3	75	37.3	
**Cough**							
No	1	1.1	0	0.0	1	0.5	0.441
Yes	88	98.9	113	100.0	201	99.5	
**Length of cough (weeks)**							
0 |-- 3 weeks	31	35.6	30	28.0	61	31.4	0.279
≥3 weeks	56	64.4	77	72.0	133	68.6	
**Productive cough**							
No	16	18.0	25	22.1	41	20.3	0.478
Yes	73	82.0	88	77.9	161	79.7	
**Length of productive cough (weeks)**							
0 |-- 3 weeks	45	52.3	60	56.1	105	54.4	0.663
≥3 weeks	41	47.7	47	43.9	88	45.6	
**Fever**							
No	47	52.8	11	9.8	58	28.9	<0.001
Yes	42	47.2	101	90.2	143	71.1	
**Length of fever (weeks)**							
0 |-- 3 weeks	67	77.0	58	53.7	125	64.1	0.001
≥3 weeks	20	23.0	50	46.3	70	35.9	
**Blood in sputum**							
No	72	81.8	72	63.7	144	71.6	0.005
Yes	16	18.2	41	36.3	57	28.4	
**Night sweats**							
No	69	77.5	47	42.0	116	57.7	<0.001
Yes	20	22.5	65	58.0	85	42.3	
**Appetite loss**							
No	39	43.8	25	22.1	64	31.7	0.001
Yes	50	56.2	88	77.9	138	68.3	
**Dyspnea**							
No	35	39.8	39	34.5	74	36.8	0.464
Yes	53	60.2	74	65.5	127	63.2	

**Legend: TB:** tuberculosis. *Fisher's exact test.

Among the 90 TB cases identified, anti-TB treatment was initiated in 79 (87.8%) cases: 48 during P1 and 31 during P2. At P2, 24 patients from the medium- and high-probability groups received TB treatment. Of the 23 patients in P2 in whom *Mycobacterium tuberculosis* growth was documented in their culture, 6 were classified as non-TB or low probability and 17 were classified as medium or high probability by the NCS-TB. Empirical treatment was provided to 12/48 (25%) patients in P1 and 7/21 (33.3%) patients in P2 (*p*=0.558). 

Among all pPTB subjects and among the HIV co-infected subgroup, the NCS-TB P1 and P2 cohorts showed a sensitivity of 76.5% and 80%, specificity of 48.2% and 37.7%, and accuracy of 55.2% and 40.9%, respectively, when compared with the combined diagnostic performance of sputum smear microscopy, sputum culture, and clinical-radiological criteria (data not shown). 

Table 2 shows the predictive values of the NCS-TB according to the accuracy observed in this study and the different TB prevalence rates in the health system. As a triage method, the NCS-TB showed a negative predictive value higher than 95% in scenarios where the TB prevalence ranged from 1% to 10%. 

**TABLE 2:** Predictive values of the Neural Clinical Score for TB according to TB prevalence.

Accuracy of the NCS-TB				
Sensitivity	95% CI*	Specificity	95% CI*	
76.5%	61.4-91.5	48.2%	40.6-55.8	
TB Prevalence**				
	1.0%	4.0%	10%	24.8%
Positive Predictive Value	1.5%	5.8%	14%	33%
Negative Predictive Value	99.5%	98%	94.9%	86%

Legend: *CI: 95% confidence interval; **TB prevalence: 1% in the family health program, 4% in the basic health unit, 10% in emergency care, and 24.8% in the present study. **NCS-TB:** Neural Clinical Score for TB; **TB:** tuberculosis.

Comparing P2 and P1, the NCS-TB shortened the time from triage to microscopy results by approximately 1 d (from 3.3 to 2.5 days, *p*<0.001) and the time to therapy initiation from 6.7 to 4.1 days (*p*=0.045).

Inadequate clinical specimen collection was observed in 12 of 147 (8.2%) samples during P1 and in 4 of 113 (3.5%) during P2.

In the intervention (P2), the NCS-TB classified 89 patients (44.1%) as having a low probability; among them, 8 (8.9%) were TB cases. A total of 113 patients (55.9%) had a medium or high probability, and among them, 26 (23%) were TB cases.

Bacteriologically-confirmed TB (smear microscopy or sputum culture) and the use of clinical-radiological criteria were the diagnostic methods performed in 78% (44/56) and 21.4% (12/56) of P1 cases and in 82% (28/34) and 17.6% (6/56) of P2 cases, respectively.

The results of this study suggest that the clinical score of the NCS-TB, without chest X-ray image analysis, had a higher negative predictive value in scenarios with a high prevalence of HIV and a TB prevalence of less than 10%. Through the identification of moderate-and high-risk presumed TB subjects, the NCS-TB was able to accelerate the sample testing, contributing to an earlier adequate clinical decision, as indicated in previous studies using artificial intelligence^7^ ^-^ ^9^.

This was the first time that the NCS-TB was evaluated in a setting with a high HIV prevalence (79.4%). In 2019, the proportion of co-infections with TB and HIV in Brazil was 11%, emphasizing that TB is the main infectious cause of death in HIV-positive individuals^10^.

An important finding was that the NCS-TB shortened two core steps in breaking the chain of TB transmission: the time between screening and laboratory direct smear results and appropriate TB therapy initiation. 

The WHO recommends the use of innovative screening or triage approaches, usually with molecular tests or pulmonary images, with a sensitivity of 90% and specificity of 70%^11^.

In this study, the NCS-TB, which analyzed only clinical and social variables, showed lower accuracy among HIV-infected subjects (80%) than previously described in immunocompetent individuals^8^; therefore, it did not meet the WHO criteria for use as a screening tool in this scenario. However, this performance is similar to that reported for a standardized screening rule-out for pulmonary TB among HIV-seropositive subjects in resource-constrained settings, which combined signs and symptoms (cough, fever, night sweating, and weight loss) had a 78.9% sensitivity and 49.6% specificity^12^. 

The original NCS-TB was developed and tested in a low-HIV prevalence setting in Rio de Janeiro, Brazil^5^. In the current study, the NCS-TB had a low (86%) negative predictive value when applied in the HIV reference hospital. However, it should also be emphasized that, in situations where the TB prevalence is lower than 10%, at the primary healthcare level^13^ and in emergency units, and even in high HIV prevalence settings, the adoption of the proposed NCS-TB may yield high negative predictive values (greater than 95%). 

In the present study, sputum smear microscopy was optimized by a clinical score, thereby providing faster TB diagnosis, as described by others^14^ in decision analysis modeling. For this reason, this score could also be a valuable tool for immigrants from countries with high TB prevalence, as performed previously in Switzerland with symptom- and history-based screening^15^.

Therefore, the NCS-TB may be a useful adjuvant approach for TB diagnosis in HIV prevalence settings in situations where sputum smear microscopy is not available and empirical treatment is usually prescribed based on clinical and radiological criteria^16^. The NCS-TB can also guide the adoption of infection control measures by identifying medium- and high-probability patients who need to wear surgical masks during their stay at the hospital. The questionnaire helped speed up the process of TB diagnosis, and its administration took only approximately 10 to 15 minutes. 

Study limitations: This study had several limitations. It included a small sample size of pPTB subjects co-infected with HIV, which may not be representative of the entire city. No data were collected on the clinical probability defined by the attending physician in the observational phase, and information concerning local patient recruitment was not discriminated in the data bank. The best clinical trial (randomized) model was not used for operational reasons. The radiological characteristics of the patients who underwent radiography were not evaluated.

In conclusion, the NCS-TB was not suitable as a screening tool for patients presumed to have pulmonary TB in a high HIV prevalence setting. It was possible, by selecting moderate-and high-risk presumed TB subjects, to optimize laboratory diagnosis and shorten the time to treatment initiation.

## SUPPLEMENTARY MATERIAL

**SUPPLEMENTARY MATERIAL 1:** Sociodemographic characteristics of the study population according to the study phase.

	Study Phase						
	Baseline		Intervention		Total		*p**
	n	%	N	%	n	%	
**Sex**							
Female	49	30.4	81	40.1	130	35.8	0.062
Male	112	69.6	121	59.9	233	64.2	
**Race (skin color)**							
White/yellow	36	22.6	39	19.3	75	20.8	0.514
Brown/black	123	77.4	163	80.7	286	79.2	
**Marital status**							
Married/Stable union	56	34.8	84	41.6	140	38.6	0.305
Divorced/separated/widower	21	13.0	29	14.4	50	13.8	
Single	84	52.2	89	44.1	173	47.7	
**Years of schooling**							
Illiterate/<4 years	36	22.4	42	20.8	78	21.5	0.949
4 to <9 years	52	32.3	69	34.2	121	33.3	
9 years	29	18.0	39	19.3	68	18.7	
≥12 years	44	27.3	52	25.7	96	26.4	
**Socioeconomic level (minimum wage)**							
≥6	8	5.0	16	7.9	24	6.6	0.086
4-5	55	34.2	86	42.6	141	38.8	
≤3	98	60.9	100	49.5	198	54.5	
**Smoker**							
No	72	44.7	83	41.1	155	42.7	0.076
Yes	42	26.1	74	36.6	116	32.0	
Ex-smoker	47	29.2	45	22.3	92	25.3	
**CAGE criteria****							
Negative	137	86.2	166	85.1	303	85.6	0.879
Positive	22	13.8	29	14.9	51	14.4	
**Illicit drug use**							
No	131	88.5	184	91.1	315	90.0	0.473
Yes/Ex-user	17	11.5	18	8.9	35	10.0	
**HIV (HIV + pre-screen and/or post-screen)**							
Negative	22	28.9	9	12.0	31	20.5	0.015
Positive	54	71.1	66	88.0	120	79.4	

**Legend: ***Fisher's exact test. ******Alcoholism was evaluated using the cut down, annoyed, guilty, and eye-opener questionnaire (CAGE)4.

## Appendix

**SUPPLEMENTARY MATERIAL 2:** Prevalence ratio of significant variables (p<0.05) for cases of pulmonary tuberculosis compared with non-cases, Fortaleza CE. March 2011 December 2012.

	OR	95% CI^¥^
**Sex**		
Female	1.000	-
Male	1.534	1.013-2.324
**Years of schooling**		
Illiterate/<4 years	1.582	0.842-2.975
4 to <9 years	1.927	1.099-3.379
9 years	2.420	1.353-4.328
≥12 years	1.000	-
**Length of productive cough (weeks)**		
0 |-- 3 weeks	1.000	-
≥3 weeks	1.769	1.199 -2.608
**Fever**		
No	1.000	-
Yes	2.262	1.316-3.888
**Length of fever (weeks)**		
0 |-- 3 weeks	1.000	-
≥3 weeks	1.729	1.196-2.499
**Night sweats**		
No	1.000	-
Yes	1.614	1.128-2.311
**Appetite loss**		
No	1.000	-
Yes	1.927	1.207-3.075
**Coryza, nose pruritus, frequent sneeze**		
No	1.972	1.314-2.959
Yes	1.000	-

**Legend:** ¥ 95% Confidence Interval.
